# Exploring the characteristics of immortalized human ovarian surface epithelial cell lines

**DOI:** 10.1016/j.heliyon.2025.e42539

**Published:** 2025-02-07

**Authors:** Ha-Yeon Shin, Wookyeom Yang, Jue Young Kim, Ha Kyun Chang, Jongman Yoo, Woo Hee Choi, Gwan Hee Han, Hanbyoul Cho, Jae-Hoon Kim

**Affiliations:** a Department of Obstetrics and Gynecology, Gangnam Severance Hospital, Yonsei University College of Medicine, Seoul, 06273, Republic of Korea; b Department of Microbiology, CHA University School of Medicine, CHA Organoid Research Center, CHA University, R&D Institute, ORGANOIDSCIENCES Ltd., Seongnam, 13488, Republic of Korea; c Department of Obstetrics and Gynecology, Ewha Womans University Seoul Hospital, 260, Gonghang-daero, Gangseo-gu, Seoul, 07804, Republic of Korea; d Department of Obstetrics and Gynecology, Sanggye Paik Hospital, Inje University College of Medicine, Seoul, 01757, Republic of Korea

**Keywords:** Epithelial ovarian cancer, Immortalized human OSE (IHOSE) cell lines, Opal multiplex-IHC, Single-cell heterogeneity

## Abstract

The origins of epithelial ovarian cancer (EOC) have long been debated, with proposed sources including ovarian surface epithelial (OSE) cells, secondary Müllerian tract structures, or fallopian tube epithelium. Despite being the second most common gynecological cancer and a leading cause of death in the United States, in vitro cell models mimicking normal ovarian epithelial cells and their malignant counterparts are lacking. To address this gap, we established immortalized human OSE (IHOSE) cell lines that demonstrate stable in vitro growth without malignant properties. IHOSE cell lines were unique cell lines by analyzing short tandem repeat (STR) profiling. In addition, the epithelial characteristics were confirmed by cytokeratin 7 and cytokeratin 18 marker expression. IHOSE cell lines were subjected to Opal multiplex immunohistochemistry (IHC) analysis, which established four distinct subtypes based on marker dominance. These studies offer the most basic but essential cellular characterization information for IHOSE cell lines, providing critical data that can guide the selection of cells when inducing normal controls or disease models.

## 1 Introduction

The ovary is a highly active organ in women of reproductive age. Women typically ovulate once a month, and the fact that the epithelial cells surrounding the ovary are only one cell layer thick, unlike those in other organs, may explain why ovulation occurs easily [1,2]. Repeated exposure to the acute proinflammatory environment that follows ovulation at the ovarian surface and within the distal fallopian tube over a woman's reproductive lifespan may contribute to an elevated risk of ovarian cancer [3]. Factors that increase the lifetime number of ovulatory cycles, such as early menarche, late menopause, nulliparity, and consistent ovulation, are associated with an increased risk of developing ovarian cancer [4].

Ovarian cancer is one of the most lethal gynecologic malignancies due to its frequent late-stage diagnosis, high recurrence rates, and chemotherapy resistance [5]. Ovarian cancer most commonly develops in the epithelial cells, which are in the outer surface tissue of the ovaries or fallopian tubes [6,7]. In fact, epithelial tumors account for 95 % of all ovarian cancer cases [8,9]. To understand the pathogenesis for EOC, research should start from the ovarian surface epithelial cells.

Efforts to establish in vitro OSE cell lines have been ongoing for decades, primarily using oncogenes such as HPV E6/E7 or SV40 T, which inhibit p53 and RB pathways. These approaches have led to the development of IHOSE cell lines, which have been characterized for their growth properties, tumorigenicity, and cytokeratin expression profiles, confirming that they retain the features of normal epithelial cells [10]. More recently, safer methods have been developed, such as the use of Sendai virus (SeV) vectors, which do not integrate into the host genome. These methods have enabled the establishment of IHOSE cell lines harboring BRCA1/2 mutations, facilitating studies on tumor formation [11]. Furthermore, with the hypothesis that high-grade serous ovarian cancer (HGSOC) originates from fallopian tube epithelial cells, immortalized fallopian tube secretory epithelial cells and fimbriae epithelial cells have been developed and are now widely used as models to study the early carcinogenic processes of ovarian cancer [12,13].

When studying a disease, it is common practice to compare its biological characteristics with those of a normal control group that is representative of a group without this disease [14,15]. In laboratory experiments, biological materials such as formalin-fixed paraffin-embedded (FFPE) or fresh frozen tissue are typically used [16,17]. Although these materials contain sufficient information about the disease, they cannot replicate the unique functions of viable cells. To overcome this, many researchers have used cell lines isolated from lesioned tissue [18], and patient-derived tissue xenograft (PDX) or organoid (PDO) models, which reflect the patient's tumor microenvironment, are valuable for preclinical studies [19]. Ovarian cancer models, such as patient-derived cell lines, PDX, and PDO, are widely used to evaluate the efficacy of anticancer drugs. Regardless of the effectiveness of all anticancer drugs, it is difficult to use them clinically if they cause significant damage to normal cells. Therefore, a control model is required to evaluate the efficacy of anticancer drugs.

In this study, we established normal ovarian epithelial cell lines as a control model for ovarian cancer cell lines and performed additional experiments, including previously reported cell lines, to determine more definitive molecular characteristics.

## 2 Materials and methods

### 2.1 Patients and ethical statement

This study was approved by the Institutional Review Board of Gangnam Severance Hospital (3-2023-0326; Seoul, Korea). Five patients were enrolled, and all experiments were conducted with the patients’ full understanding and written consent, in accordance with the Declaration of Helsinki. 5 patients' characteristics were listed in Supplementary Table 1.

### 2.2 Cell culture

HOSE cells were obtained by gently scraping the surfaces of ovaries from benign disease patients using a brush. The HOSE cells were transported to the laboratory in culture media. After centrifugation at 1000 rpm for 5 min, the supernatant was removed. The cell pellet was resuspended in fresh culture media and transferred to a culture dish. The culture media was replaced after 3 days of incubation. The monolayer of HOSE cells was cultured with Dulbecco's Modified Eagle Medium (DMEM) supplemented with 10 % Fetal bovine serum (FBS) and 1 % penicillin/streptomycin. Immortalization of HOSE cells was induced by transfecting HPV E6/E7 and SV40 T antigen to short-cultured HOSE cells using a lentiviral system following a previous report [20].

Immortalized cells were observed from 2 cell types: epithelial cells and epithelial cells with fibroblast. To isolate only epithelial cells from cells contaminated with fibroblast, we captured an epithelial cell clone in a culture dish using cloning rings (Fisher scientific, USA) and treated trypsin on the cloning ring. Trypsinased cells were transferred to 96well plates and cultured only epithelial cells.The cells were cultured in DMEM containing 10 % FBS with 1 % penicillin/streptomycin at 37 °C in 5 % CO_2_. Microscopic imaging of the cells was performed using a microscope (Life Technologies, EVOS® FL Cell Imaging System). The OVCAR3, TOV112D, and SKOV3 (RRID: CVCL_0532) cell lines were obtained from the American Type Culture Collection (ATCC, Manassas, VA). The SKOV3 and OVCAR3 cell lines were maintained in RPMI-1640 supplemented with 10 % FBS and 1 % penicillin/streptomycin, while the TOV112D cell line was cultured in DMEM containing 10 % FBS with 1 % penicillin/streptomycin at 37 °C in 5 % CO_2_.

### 2.3 Reverse transcription polymerase chain reaction (RT-PCR)

At 70–80 % confluence, the cells were washed with PBS, and total RNA was extracted using TRIzol Reagent according to the manufacturer's protocol (Ambion, Carlsbad). The Maxima First Strand cDNA Synthesis Kit (Thermo Scientific, Waltham, MA) was used to reverse transcribe 2 μg of total RNA from each sample into cDNA following the manufacturer's protocol. RT-PCR was performed with Real-taq polymerase (RBC Bioscience, New Taipei City, Taiwan) and a PCR machine (C1000 TouchTM Thermal Cycler, Bio-Rad) according to the manufacturer's instructions. The PCR products were separated on a 1 % agarose gel and detected using a Gel Doc XR + imaging system (Bio-Rad Laboratories, Inc, Hercules, CA, USA). The following primers were used in the PCR: SV40T, forward 5′- GCCCAGCCACTATAAGTACCA - 3′ and reverse 5′- CAAGCAACTCCAGCCATCCA -3'; E6, forward 5' – ATGCACCAAAAGAGAACTGCA.

AT - 3′ and reverse 5′- CAGCTGGGTTTCTCTACGTG - 3'; E7, forward 5′- ATGCATG.

GAGATACACCTACATT - 3′ and reverse 5′ ATGGTTTCTGAGAACAGATGG - 3'; β-actin, forward 5′- CTCGCCTTTGCCGATCC - 3′ and reverse 5′- GGGGTACTTCAGGG.

TGAGGA - 3'.

### 2.4 STR profiling

The genomic DNA of IHOSE cells was extracted using the Total DNA Extraction Kit (iNtRON Biotechnology, Seoul, Republic of Korea). To analyze the STR profiling, we used a cell line authentication service (Cosmo Genetech, Seoul, Republic of Korea). Genomic DNA was processed with a PCR Amplification kit (Applied Biosystems, Foster, CA) according to the manufacturer's instructions. After PCR amplification, the samples were analyzed using the ABI 3130xl Genetic Analyzer (Applied Biosystems) and the GeneMapper v5.0 software (Applied Biosystems). The STR profiling results were compared and matched using CLASTR (https://www.cellosaurus.org/str-search/).

### 2.5 Protein extraction and western blotting

Cell lysates were obtained using a cell lysis buffer (#9803 Cell Signaling Technology) supplemented with PMSF (#8553, Cell Signaling Technology). Protein concentrations were determined by BCA assay (Sigma-Aldrich, St. Louis, MO). The proteins were separated by SDS-PAGE and transferred onto 0.2 μm nitrocellulose membranes (Pall Corporation, Washington, NY). The protein bands were visualized using a western blotting luminol reagent (Santa Cruz Biotechology, Inc., Dallas, Texas) after incubation with an HRP-conjugated secondary antibody. The primary antibodies used were anti-Cytokeratin 7 (sc-23879), anti-Cytokeratin 18 (sc-515852), and anti-α-actinin (sc-17829, RRID: AB_626633) from Santa Cruz Biotechnology and anti-E-cadherin (#14472, RRID:AB_2728770), anti-EpCAM (#3599, RRID:AB_2098656), and anti-Vimentin (#5741, RRID:AB_10695459) from Cell Signaling Technology (Danvers, MA).

### 2.6 Immunofluorescence staining

Cells were seeded at 1 x 10^5^ in a 24-well plate. Cells were fixed with ice-cold methanol. The cells were incubated with anti-α-tubulin antibody (1:100, sc-5286, Santa Cruz) and then incubated with anti-Mouse IgG conjugated to Alexa Fluor 488 dye (#4408s, RRID:AB_10694704, Cell Signaling Technology). The detailed procedure was described previous report [21].

### 2.7 Growth curve and doubling time

Cells were seeded at a density of 4 x 10^4^ cells per well in a 6-well plate and cultured for 8 days, with cell counts measured every 2 days. 10 μL of resuspended cells were measured using a LUNAIITM automated cell counter (Logos Biosystems, Inc., Anyang, Republic of Korea). The doubling time of each cell line was calculated with cell counts between 2 and 8 days according to a previously described formula [20]. The growth curve was plotted using Graphpad Prism 9 (GraphPad Prism, RRID: SCR_002798), and the presented data includes means and standard deviations.

### 2.8 Soft agar assay for colony formation

Cells were seeded at a density of 1.5 x 10^5^ on 0.35 % noble agar containing 10 % FBS underneath 0.6 % top noble agar in a 6-well plate. To prevent the cell plates from drying out and to ensure sufficient nutrient supply, 500 μL of media was added every week. After three weeks, colonies were stained using a 5 mg/mL 3-(4,5-dimethylthiazol-2-yl)-2,5-diphenyl tetrazolium (MTT) solution. The cells were imaged using ZEN imaging software, which drives all ZEISS light microscope systems (Carl Zeiss Sushou.Co., Ltd).

### 2.9 Paraffin-embedded cell block array

At least 1x10^8^ cells per IHOSE cell lines were collected in a tube and fixed in 95 % ethanol. The cell pellet with 3 % agar was placed in a tissue cassette and made a paraffin-embedded cell block. One cell block array were established obtaining 3 mm core from the cell blocks of 11 IHOSE and 3 ovarian cancer cell lines.

### 2.10 Immunohistochemistry

Sectined slides were deparaffinized and rehydrated. They were retrieved in pH 6.0 citrate buffer (Agilent Technologies, Inc.,Santa Clara, CA, USA) via microwave. Then, the sections were inactivated of endogenous peroxidase with 3 % hydrogen peroxide (DAKO, #S2023), and incubated with the antibody of anti-CA-125 (Cell signaling, #19017) for 2 h. The secondary antibody was applied for 1 h, followed by detection using 3,3-diaminobenzidine tetrachloride (DAB; DAKO, #K3468) and counterstained with hematoxylin (DAKO, #S3309) for 5 min. All procedures were performed at a room temperature of 25 °C. Represenative images were captured using Microscope Axio Imager.M2 (Carl Zeiss, Thornwood, NY, Magnification ×400).

### 2.11 Opal Multiplex-IHC

The primary antibodies used were anti-PAX8 (#10336-1-AP) from Proteintech (Rosemont, USA) and anti-Cytokeratin 7 (sc-23876) from Santa Cruz Biotechnology and anti-Calretinin (#92635), anti-E-cadherin (#14472, RRID:AB_2728770), anti-MUC1 (#4538, RRID:AB_2148549), and anti-CA-125 (#19017, RRID:AB_2924774) from Cell Signaling Technology (Danvers, MA).

Multiplex immunofluorescence staining was performed using the Opal Polaris 7-Color Manual IHC Kit (Akoya Biosciences, Marlborough, MA, USA) according to the manufacturer's protocol (AKOYA). First, cell block array sections were deparaffinized in xylene and hydrated through gradually varying concentrations of ethanol from high (100 %) to low (70 %). Next, antigen retrieval was performed in Tris-EDTA pH 9.0 buffer at an output power of 1000 W using a microwave for about 70 s until the buffer boiled, then the output power was reduced to 200 W and the procedure lasted for 15 min. The first time was in AR9 buffer (Akoya Biosciences, Marlborough, MA, USA), followed by six repeated antibody stripping in AR6 buffer (Akoya Biosciences, Marlborough, MA, USA). After the antigen retrieval process, slides were washed with TBS-T (0.05 % Tween-20 in TBS) and treated with antibody diluent/block buffer (ARD1001EA, AKOYA, Marlborough, MA, USA) for 10 min at room temperature followed by 60 min of reaction with primary antibody. The antibody was diluted in Antibody Diluent/block buffer. Next, after washing three times with TBS-T, a solution of OPAL polymer HRP Ms + Rb (ARH1001EA, AKOYA, Marlborough, MA, USA) was dropped to the cell block and treated for 10 min at room temperature. Similarly, after washing with TBS-T, Opal Fluorophore is diluted 1:100 in 1xAmplification Diluent (FP1498, AKOYA, Marlborough, MA, USA) solution and reacted for 10 min at room temperature under shade. Afterward, use a microwave oven to perform antibody stripping using the same output power as the previous antigen retrieval procedure. Washing three times with TBS-T and then repeat from blocking stage in antibody diluent/block buffer. Opal Fluorophore were used to stain the sections: Opal570 (AKNEL861001KT; AKOYA, Marlborough, MA, USA), Opal520 (AKNEL861001KT), Opal620 (AKNEL861001KT), Opal690 (AKNEL861001KT), Opal480 (AKNEL861001KT), and Opal780 (AKNEL861001KT). The staining was carried out in the same way as above until the fifth fluorophore was detected; finally, for the sixth fluorophore, the Opal polymer HRP Ms + Rb solution was treated with Opal TSA-DIG stock solution diluted with 1xAmplification Diluent 1:100, followed by a 10 min treatment at room temperature under light blocking. After processing, antibody stripping was performed and Opal Polaris 780 is diluted in Antibody Diluent/Block buffer and incubated for 60 min at room temperature in the dark. After all staining is complete, dilute Spectral DAPI solution in TBS-T and react for 5 min in the dark at room temperature, followed by a brief wash and mounting with prolong diamond anti-fade mountant.

### 2.12 Analysis of imaging

Multispectral imaging at 20x magnification was performed using PhenoImager Fusion (Akoya Biosciences). The raw data was generated in Qptiff file format and the images were analyzed using QuPath (RRID:SCR_018257) quantitative pathology & bioimage analysis. A portion of the area to be analyzed is designated as a region and cell segmentation is performed using the algorithm in the program. After that, cell phenotyping is performed by classifying the cells that expressed each marker. After the cell phenotyping was completed, the detection measurement data was extracted and statistical analysis (Violin plot, Ridge plot, UMAP, pie chart) was performed using the R.studio program and code.

## 3 Results

### 3.1 The novel six IHOSE cell lines established via SV40 T or HPV E6/E7

**Fig. 1:**
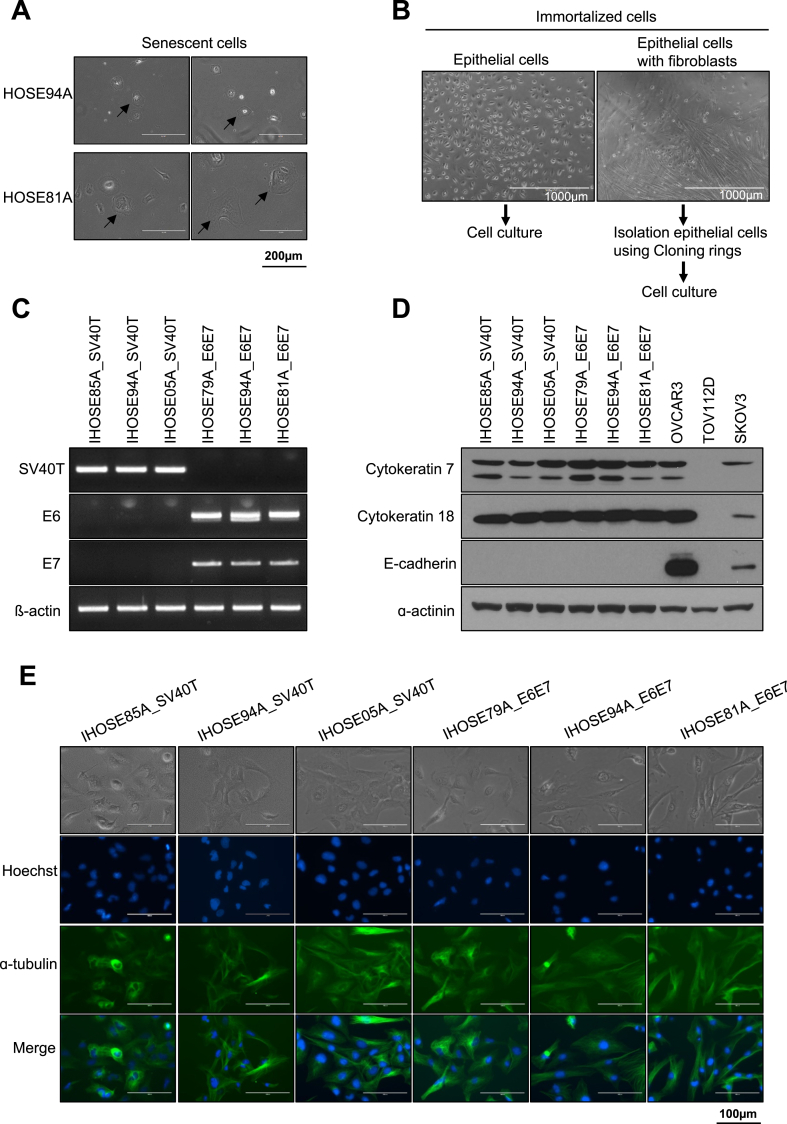
The novel six IHOSE cell lines were established via SV40 T or HPV E6/E7. (A) Representative images show the morphology of aged cells (indicated by arrows). Scale bar: 200 μm, 200× magnification. (B) After the immortalization process, the left images show that epithelial cells are cultured. The right image is epithelial cells with fibroblast, and they isolate only epithelial cells excluding fibroblast using a Cloning ring. (C) The expression levels of SV40 T antigen and HPV E6/E7 in IHOSE cells were detected by RT-PCR. β-actin was used as a loading control. (D) The expression levels of indicated protein were measured by immunoblotting in the IHOSE and cancer cell lines. ɑ-actinin was used as a loading control. (E) Cytoskeleton was detected by Immunofluorescence using an anti-ɑ-tubulin (green) antibody in six IHOSE cell lines. Nuclei are stained with Hoechst (blue). Scale bar: 100 μm, 400× magnification.

Human-derived ovarian surface epithelial cells obtained from humans were cultured until the cells stopped growing and progressed. The cells showed the typical morphology of aged cells, flattening, and a large cell size (Fig. 1A). We attempted oncogene infection of senescent cells for immortalization. HOSE cells obtained from five patients were infected with lentiviral particles expressing SV40T or HPV E6/E7 and six stably growing immortalized HOSE (IHOSE) cell lines were obtained. Primary epithelial cell cultures were carefully mixed with fibroblasts. We observed that five IHOSE cell lines cultured only epithelial cells, whereas IHOSE94A_SV40T showed contamination with fibroblasts. Only the epithelial cells were separated using a cloning ring (Fig. 1B). Six IHOSE cell lines stably expressed SV40T and HPV-E6/E7 mRNA (Fig. 1C). Immunoblotting established that the epithelial cell markers cytokeratin 7 and cytokeratin 18 were strongly expressed in all IHOSE cell lines. However, epithelial marker E-cadherin was did not observed in IHOSE cell lines (Fig. 1D). The morphology of the growing six IHOSE cell lines is shown in Fig. 1E. We confirmed the absence of aged cells. Immunofluorescence images distinguished the nucleus and cytoskeleton of IHOSE cell lines using dyed Hoechst and ɑtubulin (Fig. 1E).

Genomic DNA was extracted from six IHOSE cell lines and DNA fingerprinting was performed using 18 STR loci. A comparison of 18 STR loci profiles based on the CLASTR cell line STR database revealed unique IHOSE cell lines. In addition, neither multi-allele nor genome cross-contamination was detected in the STR analysis (Table 1). IHOSE94A_E6/E7 and IHOSE94A_SV40 cells share the common HOSE progenitor cell, HOSE94A. The STR profiles of the two cell lines were identical. Six IHOSE cell lines were established.

**Table 1:** STR authentication in six IHOSE cell lines.

Locus	IHOSE85A_SV40T	IHOSE94A_SV40T	IHOSE05A_SV40T	IHOSE79A_E6E7	IHOSE94A_E6E7	IHOSE81A_E6E7
*D5S818*	11	12, 14	10, 11	9, 10	12, 14	11
*D13S317*	8, 11	8, 12	8, 10	11, 12	8, 12	9, 11
*D7S820*	8, 10	10, 12	11, 12	12, 13	10, 12	8, 11
*D16S539*	10, 12	11, 12	11	9, 11	11, 12	9, 10
*vWA*	14, 17	18, 19	18, 19	16, 17	18, 19	15, 17
*TH01*	6, 7	9, 9.3	6, 7	9	9, 9.3	6, 9
*TPOX*	8	8, 9	11	8, 11	8, 9	8, 11
*CSF1PO*	10, 11	10, 12	12	11, 12	10, 12	11, 12
*AMEL*	X	X	X	X	X	X
*D3S1358*	14, 16	17	15, 17	14, 16	17	16
*D21S11*	30, 32.2	28, 32.2	29	30, 31	28, 32.2	28.2, 31
*D18S51*	14, 17	17, 20	13, 14	13, 22	17, 20	15
*D8S1179*	14	14	11, 14	12, 15	14	14, 15
*FGA*	21, 24	23	18, 21	20, 22	23	21, 22
*D2S1338*	18, 22	20, 24	22, 24	19, 23	20, 24	17, 24
*D19S433*	13, 15	13, 14	13	14	13, 14	14, 15.2
*Penta D*	9, 10	9, 12	10, 11	12, 13	9, 12	9, 11
*Penta E*	8, 19	15, 18	15, 16	5, 23	15, 18	11, 18

### 3.2 Maintained cell morphology of IHOSE for ten passages

**Fig. 2:**
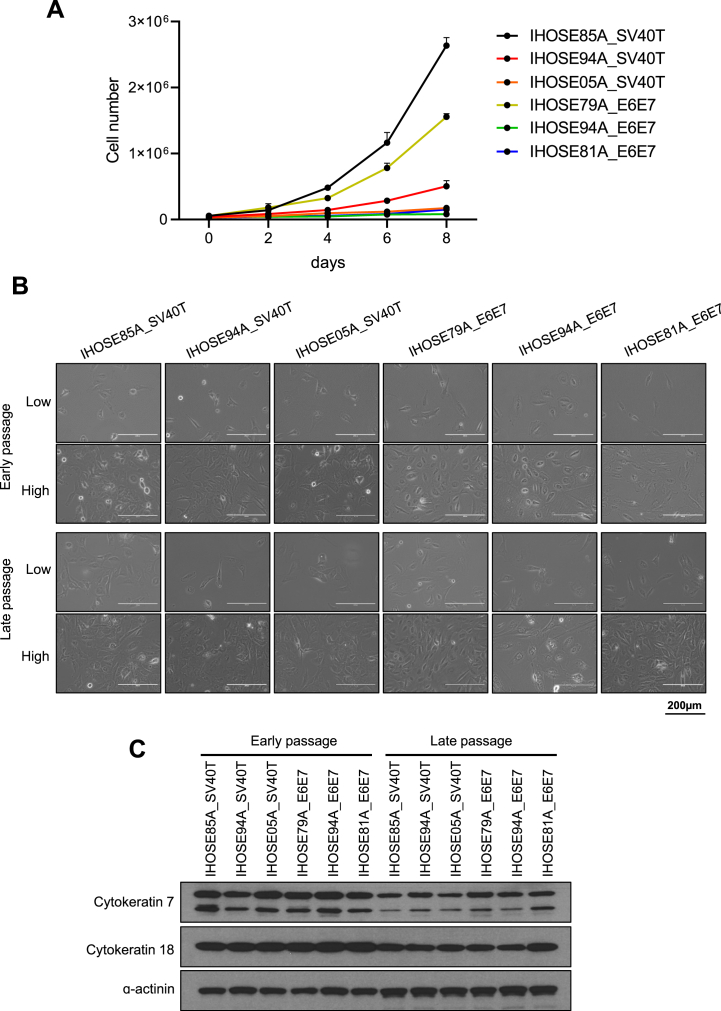
Maintained cell morphology of IHOSE for ten passages (A) Growth curves and doubling time of cells were determined by counting the cells every 2 days for 8 days. Error bars represent mean ± SD. (B). Representative images showed high and low-density cell morphology from six IHOSE cell lines. The upper panel shows the early passage of cells; the lower panel shows the late passage of cells. Scale bar: 200 μm, 200 × magnification. (C). The expression levels of indicated protein were measured by immunoblotting in the IHOSE cell lines of early and late passages. ɑ-actinin was used as a loading control.

To assess the proliferation rates of the six IHOSE cell lines, we counted the cells for up to 8 days (Fig. 2A). We found that the IHOSE85A_SV40T and IHOSE79A_E6E7 cell lines had faster doubling times than the other IHOSE cell lines at 34.71 h and 39.41 h, respectively. Conversely, the IHOSE94A_E6E7 cell line grew much slower, with a doubling time of 248.7 h. Table 2 shows the doubling time for each cell line. In our study, we established IHOSE94A_SV40T and IHOSE94A_E6E7 cell lines from the same patient using either SV40T or E6/E7. The SV40T cell line demonstrated a significantly higher proliferation rate compared to the E6/E7 cell line, likely due to SV40T's simultaneous and more efficient inhibition of both p53 and Rb, compared to the independent actions of E6 and E7 [22,23]. The IHOSE cells consistently proliferated and reached more ten passages. We captured cell morphology images at different passage numbers and cell densities (Fig. 2B), with early passage images presenting cells of two to five passages, and late passage images presenting cells of over ten passages. Notably, the six late-passage IHOSE cell lines maintained the same cell morphology as the early passages. The continuous expression of epithelial markers was confirmed by immunoblotting for cytokeratins 7 and 18 (Fig. 2C).

**Table 2:** The doubling time of six IHOSE cell lines.

Cell line	Doubling Time (h)	Standard. Deviation (S.D)
IHOSE85A_SV40T	34.71	0.40
IHOSE94A_SV40T	52.05	3.26
IHOSE05A_SV40T	72.85	5.90
IHOSE79A_E6E7	39.41	0.35
IHOSE94A_E6E7	248.70	76.56
IHOSE81A_E6E7	82.59	8.46

### 3.3 Non-malignancy characteristics of IHOSE cell lines

**Fig. 3:**
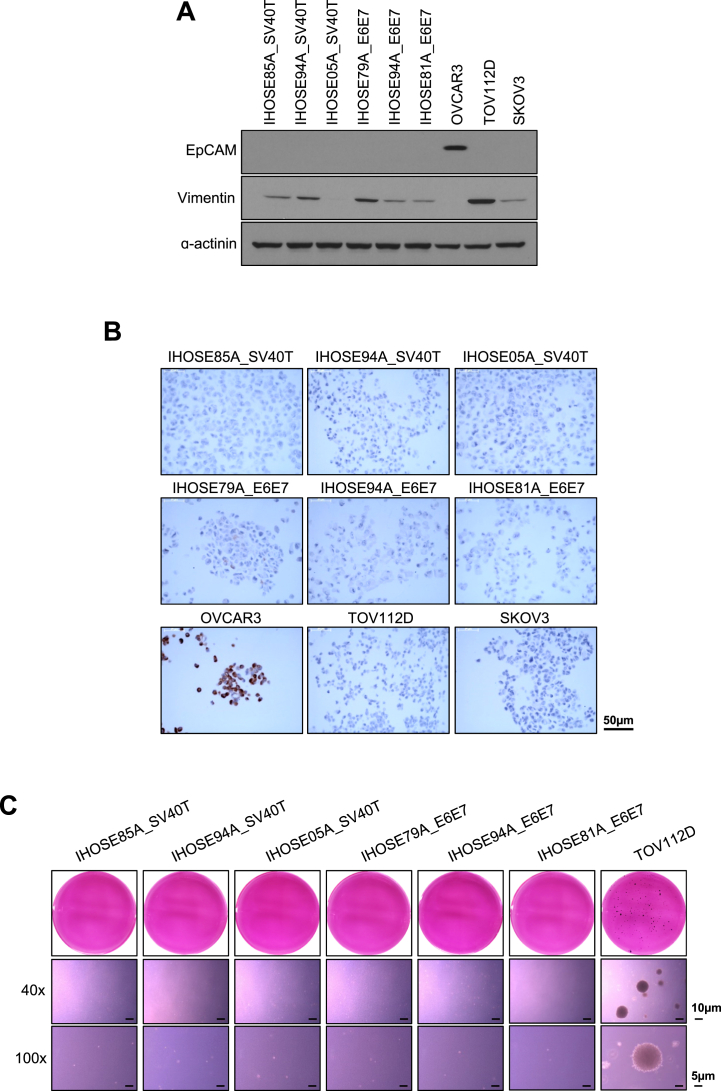
Non-malignancy characteristics of IHOSE cell lines. (A) The expression levels of indicated protein were measured by immunoblotting in the IHOSE and cancer cell lines. ɑ-actinin was used as a loading control. (B) The expression levels of CA-125 were confirmed via immunohistochemistry staining in the IHOSE and cancer cell lines. Scale bar: 50 μm. (C) Anchorage-independent soft agar assay performed in IHOSE and TOV112D cell lines. Colonies were stained by MTT solution. Representative images of colonies captured with 40x and 100x magnification. Scale bar: 10 μm and 5 μm, respectively.

Immortalization due to oncogenic infections may lead to the acquisition of malignant characteristics. We confirmed whether IHOSE cell lines exhibited malignant features by immunoblotting for cancer stem cell markers and an anchorage-independent soft agar assay. The cancer stem cell markers epithelial cell adhesion molecule (EpCAM) are overexpressed in ovarian cancer. Immunoblotting showed that the six IHOSE cell lines did not express EpCAM. In contrast, OVCAR3, an ovarian cancer cell line, showed high expression levels of EpCAM (Fig. 3A). Vimentin, an epithelial-mesenchymal transition (EMT) marker, was detected in IHOSE cells. However, vimentin, a mesenchymal intermediate filament, is co-expressed with keratin in OSE cells because most epithelial cells respond only to wounding, explantation into culture, or pathological conditions. Additionally, vimentin expression was observed both in situ and in cultured primary and passaged cells as reported in publications. The expression of vimentin in IHOSE is not due to the induction of EMT according to the immortalization process but is considered to remain an expression in primary cells [2]. To observe cancer antigen 125 (CA125) as a marker of ovarian cancer, we performed immunohistochemistry on IHOSE cell blocks. Six IHOSE cell lines did not express CA125. In contrast, OVCAR3 showed high expression of CA125 (Fig. 3B).

Another feature of cancer cells is their ability to grow in suspension. Using an anchorage-independent soft agar assay, we demonstrated that IHOSE cells did not form colonies, whereas ovarian cancer cells formed colonies (Fig. 3C). Therefore, we concluded that IHOSE cells did not exhibit the carcinogenic characteristics of ovarian cancer cells, but still exhibited the desired property of continuous cell division.

### 3.4 Analysis of single-cell levels in 11 IHOSE and 3 ovarian cancer cell lines

**Fig. 4:**
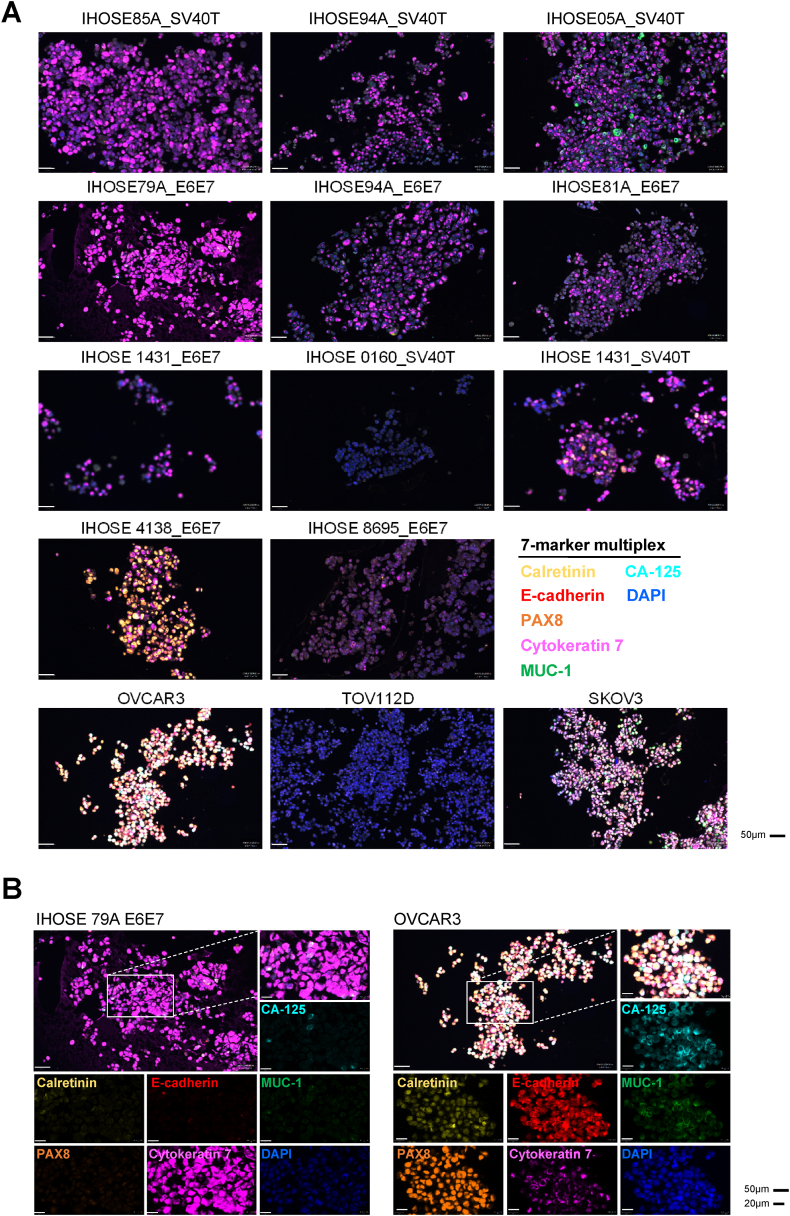
7-Color Multiplex IHC to visualize IHOSE and ovarian cancer cell lines in FFPE sections of the cell block. (A) The cell lines were analyzed in 7-marker panels in one FFPE section, showing the colocalized image of 7-markers. Biomarkers and colors are shown on the right. Scale bar: 50 μm. (B) Two cell lines represented the colocalized images of the 7-marker and corresponding single-color images; 50 μm and 20 μm, respectively.

We established and characterized 11 IHOSE cell lines, five of which have been previously reported, and six of which have been reported in this study. To further characterize cell-to-cell heterogeneity, we created one paraffin-embedded cell block, including all IHOSE cell lines and three ovarian cancer cell lines, and performed Opal multiplex immunohistochemistry. This technique allows multiple biomarkers to be simultaneously stained in one section, which helps elucidate the spatial relationships among individual cell types [24,25]. One cell-block slide section was stained for a 7-color panel containing six biomarkers (Calretinin, E-cadherin, MUC-1, PAX8, Cytokeratin 7, CA125, and DAPI). The cell lines were visualized at the single-cell level using 7-color multiplex IHC. Images of all seven markers are shown in Fig. 4A, and representative images of the IHOSE 79A_E6E7 and OVCAR3 cell lines are presented. In Supplementary Table 2, the staining intensity was categorized into four levels.

**Fig. 5:**
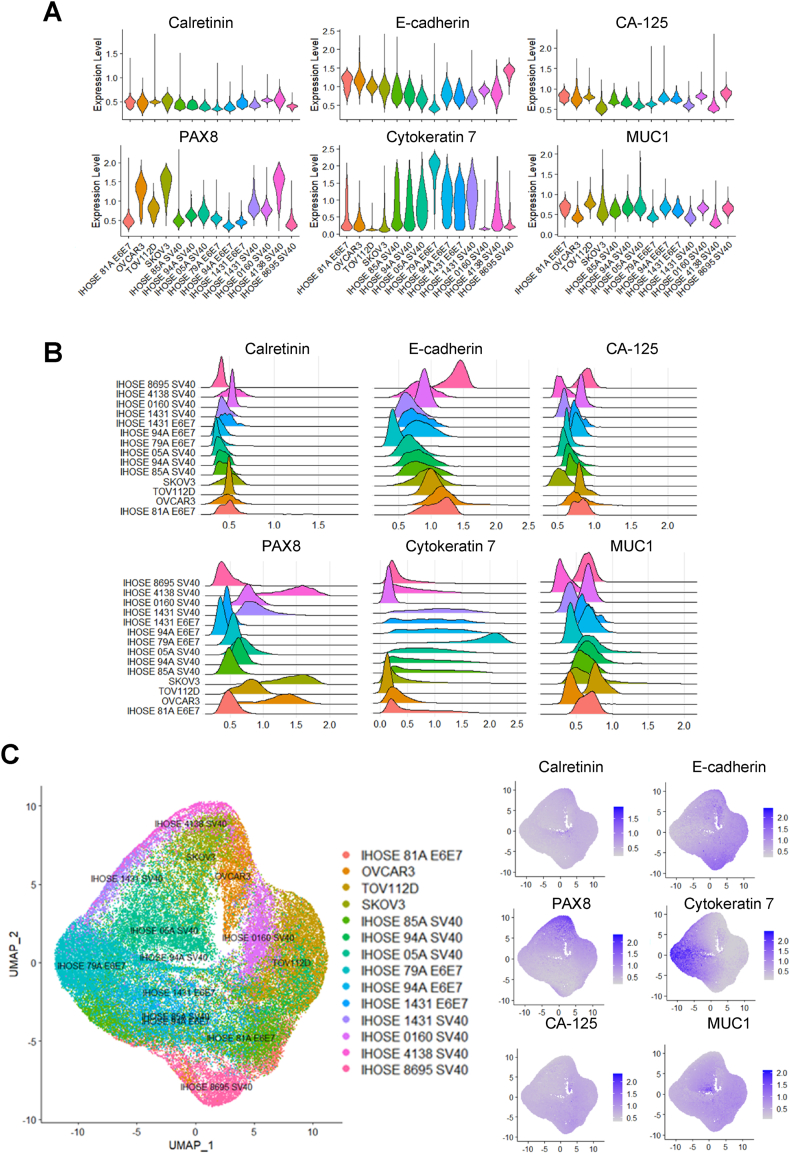
Single-cell populations in IHOSE and ovarian cancer cell lines. Violin (A) and ridge (B) plots depict the expression levels of each marker. (C) UMAP plot showing single-cell populations of IHOSE and ovarian cancer cell lines.

Violin and ridge plots of single-cell data were used to determine the expression distributions of the six markers. Among these, PAX8 expression was high in ovarian cancer cell lines, whereas cytokeratin 7 expression was high in IHOSE cell lines. The expression distribution of the remaining markers did not differ significantly between cell lines. (Fig. 5A and B). Using Uniform Manifold Approximation and Projection (UMAP), a dimension-reduction and visualization method, we determined whether single-cell heterogeneity was present according to marker expression in IHOSE and Ovarian cancer cell lines. IHOSE4138_SV40T and IHOSE0160_SV40T were located closer to the ovarian cancer cell lines than the other IHOSE cell lines (Fig. 5C). However, other IHOSE cell lines are distinct from ovarian cancer cell lines.

**Fig. 6:**
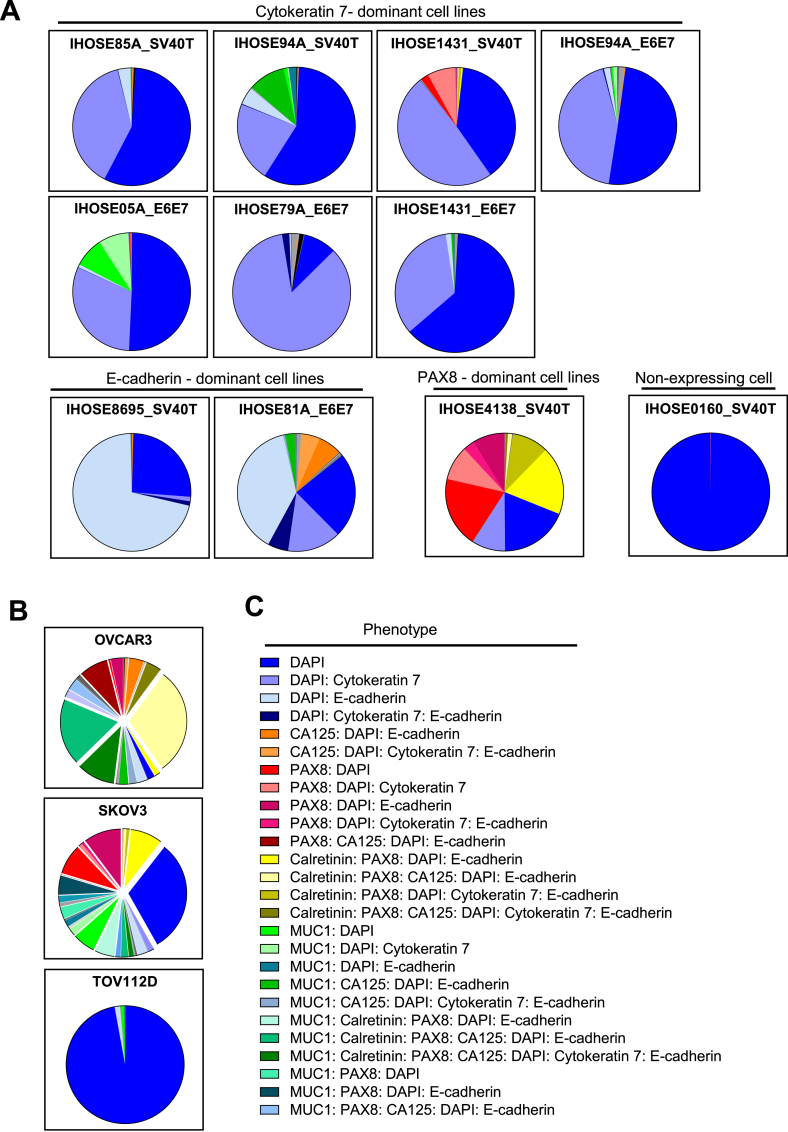
The pie chart displays the proportions of phenotype in each cell line. Pie-chart showing the phenotypes of IHOSE (A) and ovarian cancer cell lines(B). (C) Among the 57 phenotypes, only those accounting for >2 % are indicated.

We classified the cells into 57 phenotypes according to the positive cell ratios of the six markers, excluding DAPI (Supplementary Table 3). The proportions of phenotypes in IHOSE and ovarian cancer cells are displayed as a pie chart. In this study, the 11 IHOSE cell lines were divided into four types. The first type is a high proportion of cells expressing cytokeratin 7, which includes 7 of the 11 IHOSE cell lines. The second type is characterized by a high proportion of cells expressing E-cadherin and contains two IHOSE cell lines. The third type comprised a high proportion of cells expressing PAX8, which was observed in the IHOSE4138_SV40T cell line. Finally, the fourth type consisted of cells with almost no marker expression, as was the case for the IHOSE0160_SV40T cell line (Fig. 6A). Although IHOSE cell lines are classified into four types, they have a significantly monotonous cellular composition compared to that of OVCAR3 and SKOV3 cells (Fig. 6A and B).

## 4 Discussion

This study extends previous research by establishing IHOSE cell lines [20] and analyzing the expression patterns of specific proteins at the single-cell level to understand their properties.

Historically, bulk RNA sequencing has been used to measure average gene expression values by pooling all cells. However, recent trends favor single-cell sequencing, which allows the precise measurement of gene expression at the individual cell level. Under heterogeneous conditions such as in tumor cells, group-level gene expression may not fully represent the diversity of individual cells. This has led to the widespread use of spatial multiomics, which identifies the mRNA and protein expression of genes at the single-cell level and provides spatial information within the tissue [26,27].

In this study, multiplex-IHC for six markers, cytokeratin 7, E-cadherin, MUC-1, Calretinin, and PAX8, which are epithelial cell markers associated with the female genital tract and are involved in the structure and development of the cell, and the ovarian cancer marker CA-125 was applied to IHOSE cell lines [2,28]. Based on the single-cell analysis, we classified the 11 IHOSE cell lines into four different phenotypes.

As a representative epithelial cell marker, cytokeratin 7 showed prominent expression in IHOSE cell lines, as validated by western blotting and multiplex-IHC. The difference in the expression of cytokeratin 7 between cell lines categorized as cytokeratin 7-dominant, which was difficult to distinguish by western blotting, could be distinguished by single-cell resolution using multiplex-IHC. This difference in expression was confirmed using E-cadherin. Western blotting identified expression only in OVCAR3 and SKOV3 cancer cell lines, and not in IHOSE cells. However, multiplex-IHC, which employs high antibody concentrations and advanced signal amplification mechanisms such as Tyramide Signal Amplification (TSA) [29], offers very high sensitivity. This technique enabled the detection of low E-cadherin expression levels, allowing us to identify E-cadherin-dominant cells within the IHOSE cell line through multiplex-IHC. In normal cells, E-cadherin is primarily a protein that contributes to the maintenance of cell adhesion between epithelial cells [30]. According to a previous report, the induction of E-cadherin expression in immortalized OSE restored the properties of OSE that had been lost in culture. In other words, the increase in E-cadherin helped cell adhesion bonds form, resulting in a decrease in mesenchymal properties and an increase in epithelial properties [31]. Therefore, E-cadherin-dominant cell lines are thought to have different properties from those without E-cadherins.

The PAX8-dominant IHOSE4138_SV40T cell line is exciting, exhibiting high expression levels of PAX8 and calretinin, and the most diverse marker expression phenotype of individual cells. Calretinin is a calcium-binding protein known as a marker of mesothelial cells, and is commonly expressed in the ovarian surface epithelium and epithelial-containing cysts [32]. PAX8 is a major protein expressed by the fallopian tube secretory epithelial cells and is essential for embryonic development and normal function in the female reproductive system. In the ovarian surface epithelium, PAX8 is expressed in 44 % of the cases. The epithelial cells lining the ovary show differential marker expression depending on the region. As previously reported, immunostaining for calretinin, PAX8, and E-cadherin in the ovarian surface epithelium was performed by zoning, and the expression patterns differed between the zones. Therefore, it is difficult to assume that the expression patterns of epithelial cells observed in some zones are representative of the entire population of ovarian epithelial cells. Therefore, although limited sampling in our study made it difficult to represent the entire ovarian epithelial cell population, IHOSE4138_SV40T is considered a rare cell line that comprehensively demonstrates the overall diversity of ovarian epithelial cells.

The IHOSE0160_SV40T cell line showed negligible expression of epithelial cell markers, similar to the ovarian cancer cell line TOV112D. However, the two cell types were distinguishable in tumorigenicity assays. Six markers were insufficient to distinguish between the two cell types, and further research is needed.

In a recent report, the normal human endometrial epithelial cell line, hEM3, was established through the induction of SV40T, analogous to our methods. This cell line, when subjected to ARID1A knockout, has been effectively utilized for drug screening within an ARID1A-deficient and MMR-proficient context. These findings suggest that hEM3 serves as a robust model for studying the pathogenesis of endometrial cancer [33]. OSE has been hypothesized as a potential origin for low-grade serous ovarian cancer and some borderline tumors [34], providing a basis for its use in ovarian cancer research. While the role of OSE in the pathogenesis of HGSOC remains debated, OSE-derived cell lines offer key advantages. They serve as valuable models for studying early tumorigenesis, enabling the investigation of molecular and cellular changes induced by environmental or genetic stimuli. Although most HGSOCs are believed to originate from secretory cells of the fallopian tube epithelium [7], OSE is an important model due to its potential involvement in HGSOC development under specific conditions, such as inflammation or genetic predisposition, and its close interactions with the tumor microenvironment [35]. Additionally, as OSE interacts directly with ovarian stroma, immune cells, and follicular fluid, OSE-based models are particularly useful for exploring the interplay between the tumor microenvironment and cancer progression [36,37]. Therefore, by introducing genetic mutations with EOC into the IHOSE cell lines, we will create a valuable tool for investigating the mechanisms of EOC initiation and progression.

In conclusion, the application of multiplex-IHC and single-cell analysis allowed for the detailed characterization of epithelial marker expression, revealing distinct phenotypes within the IHOSE cell lines that may reflect the cellular heterogeneity observed in ovarian tissues. Markers such as Cytokeratin 7, PAX8, and E-cadherin provide critical insights into various aspects of ovarian cancer research. For instance, Cytokeratin 7-expressing cells serve as models for studying the early transition from normal epithelium to cancer cells, while PAX8-expressing cells contribute to understanding cancers originating from fallopian tube epithelium. Additionally, E-cadherin-expressing cells are valuable for investigating EMT and metastasis. Although marker expression can vary substantially between cell lines, this variability highlights the importance of selecting appropriate models tailored to specific research objectives. This study provides foundational yet essential cellular characterization data for IHOSE cell lines, deepening our understanding of ovarian cancer pathogenesis and offering a robust basis for their use as normal controls or disease models.

### 4.1 Limitations of the study

SV40T and E6/E7 remain widely used for the induction of immortalization due to their efficiency. However, recent advancements have led to the development of safer and more efficient methods to overcome the limitations associated with these traditional approaches. In earlier methods, foreign genetic material was integrated into the host genome to induce immortalization, which carried potential risks, such as genomic instability. In contrast, recent studies have adopted RNA virus-based systems that act exclusively in the cytoplasm, bypassing the host genome [11]. This approach significantly enhances safety by eliminating the risk of genomic integration while maintaining high efficiency. Our research group is also actively working to leverage these cutting-edge technologies to overcome the limitations of conventional methods and develop safer immortalized cell lines.

We attempted to categorize IHOSE cell lines based on their expression patterns using multiplex IHC. While detailed expression changes were observed at the single-cell level, we were unable to detect whether these categories represent any physiological differences. This limitation suggests that further investigation is needed to determine the functional relevance of the observed expression patterns.

## CRediT authorship contribution statement

**Ha-Yeon Shin:** Writing – review & editing, Writing – original draft, Investigation, Data curation, Conceptualization. **Wookyeom Yang:** Visualization, Validation, Methodology. **Jue Young Kim:** Writing – review & editing. **Ha Kyun Chang:** Writing – review & editing. **Jongman Yoo:** Visualization, Methodology. **Woo Hee Choi:** Visualization, Methodology. **Gwan Hee Han:** Methodology. **Hanbyoul Cho:** Methodology. **Jae-Hoon Kim:** Writing – review & editing, Supervision, Project administration, Conceptualization.

## Ethics approval and consent to participate

This study was approved by the Institutional Review Board of Gangnam Severance Hospital (3-2023-0326; Seoul, Korea). All samples were used as per the institutional ethics committee approval.

## Declaration of competing interest

The authors declare that they have no known competing financial interests or personal relationships that could have appeared to influence the work reported in this paper.
