# Supplementary material for: Exploring the characteristics of immortalized human ovarian surface epithelial cell lines
Source: Heliyon. 2025 Feb 7;11(4):e42539. doi: 10.1016/j.heliyon.2025.e42539 (PMC11874562; doi:10.1016/j.heliyon.2025.e42539)
Supplement: Multimedia component 1 [file mmc1.docx]

Supplementary Table 1. Characteristics of patients

| **Cell lines** | **Age** | **Name of operation** | **Diagnosis** |
| --- | --- | --- | --- |
| IHOSE85A_SV40T | 50 | Salpingo-oophorectomy | Mucinous cystadenoma |
| IHOSE94A_SV40T IHOSE94A_E6E7 | 46 | Salpingo-oophorectomy | Mature cystic teratoma |
| IHOSE05A_SV40T | 55 | Laparoscopic hysterectomy | Adenomyosis |
| IHOSE79A_E6E7 | 42 | Cystectomy and salpingectomy | Corpus luteal cyst Follicular cyst |
| IHOSE81A_E6E7 | 40 | Myomectomy | Leiomyoma |

Supplementary Table 2. Staining Intensity of Each Marker

| **Cell lines** | **Calretinin** | **E-cadherin** | **CA125** | **PAX8** | **Cytokeratin 7** | **MUC1** |
| --- | --- | --- | --- | --- | --- | --- |
| IHOSE85A_SV40T | + | + | + | + | ++ | + |
| IHOSE94A_SV40T | + | + | + | + | + | + |
| IHOSE05A_SV40T | + | + | + | + | ++ | + |
| IHOSE79A_E6E7 | + | + | + | + | +++ | + |
| IHOSE94A_E6E7 | + | + | + | + | ++ | + |
| IHOSE81A_E6E7 | + | ++ | + | + | + | + |
| IHOSE1431_E6E7 | + | + | + | + | ++ | + |
| IHOSE0160_SV40T | + | + | + | + | - | + |
| IHOSE1431_SV40T | + | + | + | ++ | ++ | + |
| IHOSE4138_SV40T | + | ++ | + | +++ | + | + |
| IHOSE8695_SV40T | + | ++ | + | + | + | + |
| OVCAR3 | + | +++ | ++ | +++ | + | + |
| TOV112D | + | + | + | + | - | + |
| SKOV3 | + | ++ | + | +++ | + | ++ |

The staining intensity was categorized into four levels within the range of 0 to 100 as follows:

-: Staining intensity between 0 and less than 5

+: Staining intensity between 5 and less than 25

++: Staining intensity between 25 and less than 50

+++: Staining intensity between 50 and 100, inclusive
